# Transcription, structure, and organoids translate time across the lifespan of humans and great apes

**DOI:** 10.1093/pnasnexus/pgad230

**Published:** 2023-07-20

**Authors:** Christine J Charvet, Kwadwo Ofori, Carmen Falcone, Brier A Rigby Dames

**Affiliations:** Department of Anatomy, Physiology and Pharmacology, College of Veterinary Medicine, Auburn University, 1130 Wire Road, Auburn, 36832, AL, USA; Department of Biology, Delaware State University, 1200 N. Dupont Highway, Dover, DE, 19901, USA; Department of Neuroscience, International School for Advanced Studies (SISSA), Via Bonomea, 265, 34136 Trieste, Italy; Department of Computer Science, University of Bath, Claverton Down, Bath, BA2 7AY, UK; Department of Psychology, University of Bath, Claverton Down, Bath, BA2 7AY, UK

## Abstract

How the neural structures supporting human cognition developed and arose in evolution is an enduring question of interest. Yet, we still lack appropriate procedures to align ages across primates, and this lacuna has hindered progress in understanding the evolution of biological programs. We generated a dataset of unprecedented size consisting of 573 time points from abrupt and gradual changes in behavior, anatomy, and transcription across human and 8 nonhuman primate species. We included time points from diverse human populations to capture within-species variation in the generation of cross-species age alignments. We also extracted corresponding ages from organoids. The identification of corresponding ages across the lifespan of 8 primate species, including apes (e.g., orangutans, gorillas) and monkeys (i.e., marmosets, macaques), reveals that some biological pathways are extended in humans compared with some nonhuman primates. Notably, the human lifespan is unusually extended relative to studied nonhuman primates demonstrating that very old age is a phase of life in humans that does not map to other studied primate species. More generally, our work prompts a reevaluation in the choice of a model system to understand aging given very old age in humans is a period of life without a clear counterpart in great apes.

**Significance Statement**

What is special about the duration of human development and aging has been an enduring source of interest. A significant hurdle in identifying which biological programs are unusually extended in humans is the lack of standardized approaches with which to align ages across species. We harnessed temporal variation in behavior, transcription, and anatomy to align ages across the lifespan of primates. These data reveal which biological programs are conserved, and which are modified. Harnessing time points across scales of study guides the choice of model systems to understand disease progression and can be used to enhance the care of great apes, many of which are critically endangered.

## Introduction

Humans differ in many aspects from other primates, though it is still unclear how biological programs have been modified in the human lineage. We know that developmental programs—such as synaptogenesis or brain growth—occur for an extended time in humans relative to primates (1–7). Biological changes and diseases that emerge in old age—such as brain atrophy or Alzheimer's disease—have traditionally been elusive in nonhuman primates. This elusivity is either because these diseases are largely unique to humans or because they do not live sufficiently long lifespans for the disease to manifest (8–10). A major hurdle in assessing which biological programs are conserved and which have been modified in primates is the lack of a standardized approach with which to align ages across species (11). In the present study, we generated tools for cross-species age alignments and we identified conserved and modified biological programs across primates.

The lack of great ape samples available for study has been a considerable impediment in the study of human brain evolution. One solution to this problem has been to engineer organoids from human and great ape cells to identify conserved and modified developmental programs (12–14). Many but not all studies have converged on the finding that the pace of cell maturation is slower in humans than it is in nonhuman primates (15, 16). Whether cross-species variation in organoid maturational timelines relates to individuals remains an open question (17). Here, we integrate maturational rates of brain development across cellular and organismal scales to generate age alignments.

We captured a conspicuous number of time points (i.e. 573) by aligning temporal changes in biological programs across multiple scales. The integration of time points from structural, behavioral, and transcriptional variation overcomes the challenges of small sample sizes that are typical of primate studies. We used these data to identify corresponding ages across the lifespan of human and nonhuman primate species (18, 19). In doing so, we implemented machine learning models to generate corresponding ages (20, 21). We also included time points from diverse human populations and great apes living in different conditions (e.g., captive versus wild) to capture within-species variation in extrapolated time points (22, 23). Comparative analyses of survival rates across diverse human populations show that the human lifespan is unusually extended compared with studied great apes.

## Results

### The dataset to translate ages across the lifespan

**Fig. 1.**
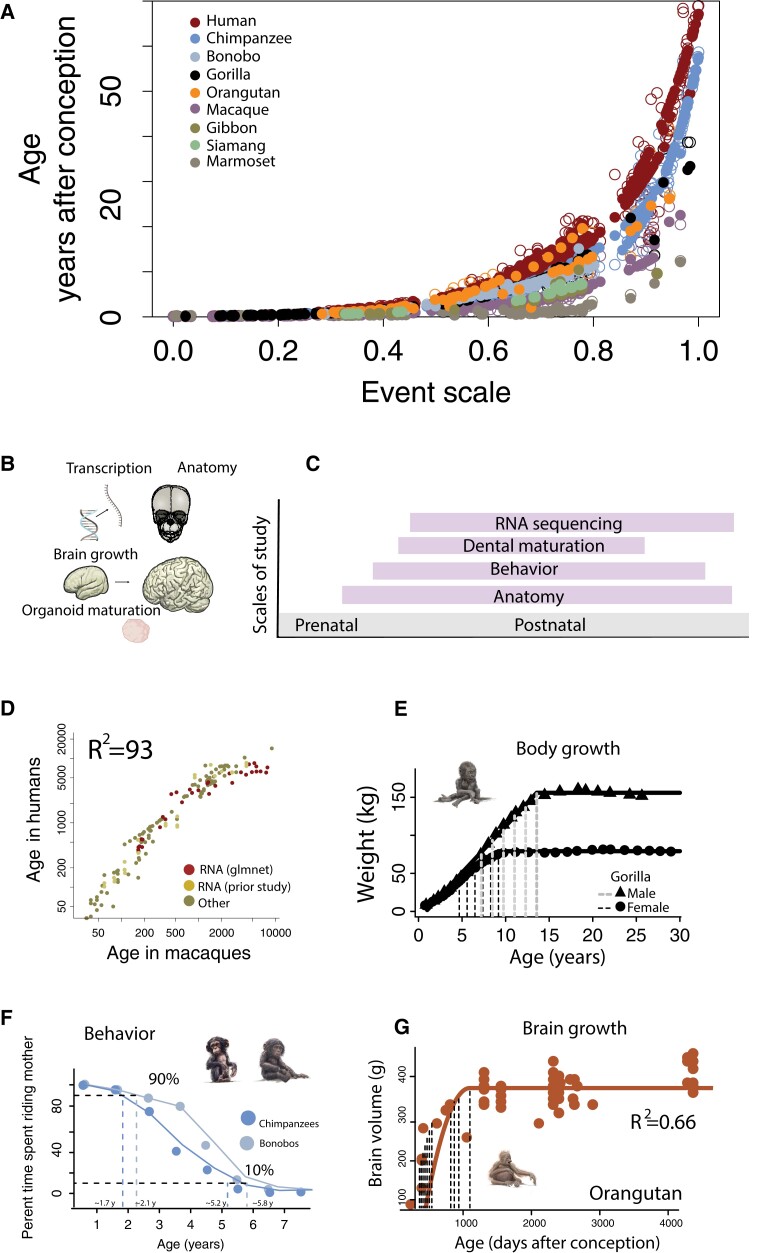
A) We developed a model to find corresponding ages across species. Predicted (closed circles) and time points (open circles) are regressed against an ordering of time points (the event scale). B) We used several metrics spanning a wide age range with different metrics represented on a life timeline. D–G) We illustrate a few examples. D) We captured time points from machine learning models from transcriptional variation. E) We also extracted time points from nonlinear regressions applied to body growth as shown for a gorilla. We captured when the body reaches a percentage of adult volume (e.g., 100% and 90%; vertical bars). We also fit smooth splines (F) through time spent riding mothers in chimpanzees and bonobos, and we quantified when the values reach a percentage of time spent riding. G) We also considered brain growth and collected when the brain reaches percentages of adult volume (vertical bars) as shown in orangutans.

**Fig. 2.**
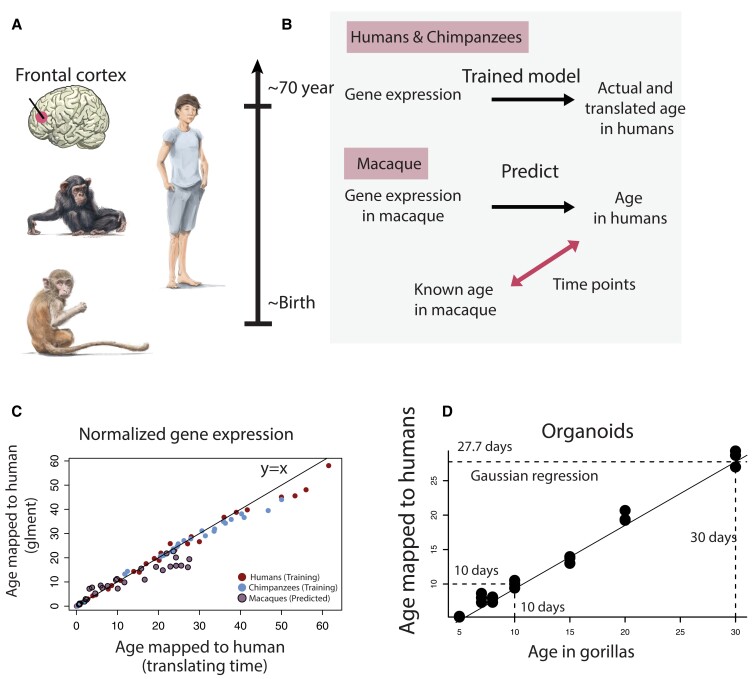
A) We generated cross-species age alignments from frontal cortex normalized gene expression in humans, chimpanzees, macaques, and organoids. B) We trained the model to predict age in a set of species. We selected the model yielding the highest age prediction accuracy (RMSE, root mean square error). We then used the selected trained model to predict age based on normalized gene expression from individuals of a different species. Because the model had been trained to predict age in humans, the model predicted ages translated to humans. We used known ages of nonhuman primates and those translated to humans as time points in the model. We include examples of age alignments from tissue (C) and from brain organoids (D).

**Fig. 3.**
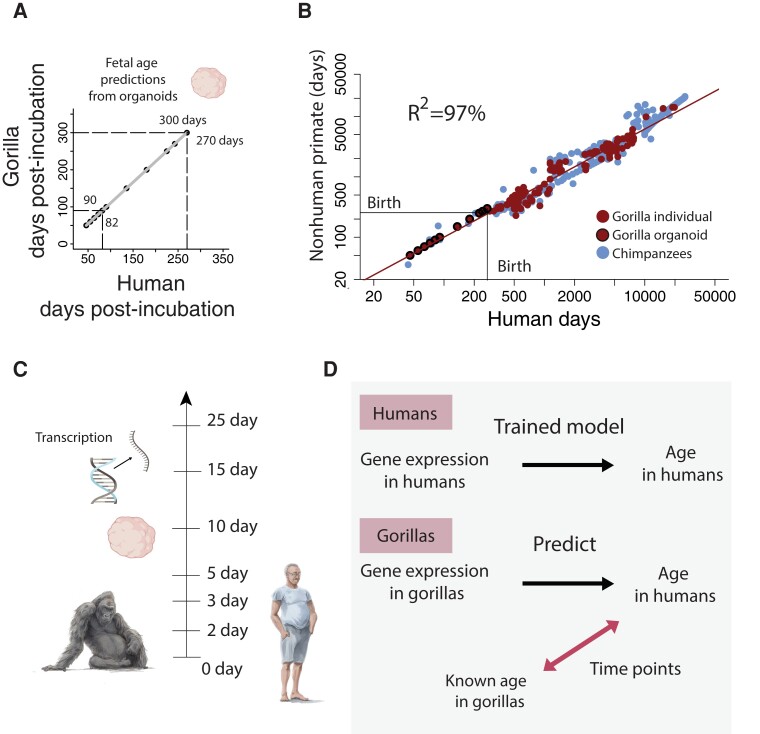
A) We identified corresponding ages between human and gorilla organoids. B) Cross-species age alignments from organoids were similar to those extracted from individuals. C) We used cerebral organoid normalized gene expression extracted from distinct individuals varying from 0 to 25 days after incubation onset (see Table S2). We used 7 different maturational states from both humans and gorillas to train machine learning models to align ages across the two species. D) We first trained models to predict age in humans from human organoid gene expression. We then used this trained model, imported normalized gene expression collected from gorillas, and predicted age. The consequence of importing data from normalized gene expression in gorillas in these models, which had been trained in humans, is that the model outputs human age (i.e. age translated from gorillas to humans). We used the age of gorilla organoids and the age of human organoids as a basis with which to translate ages across species. B) Time points collected from organoids align with time points collected from individuals.

**Fig. 4.**
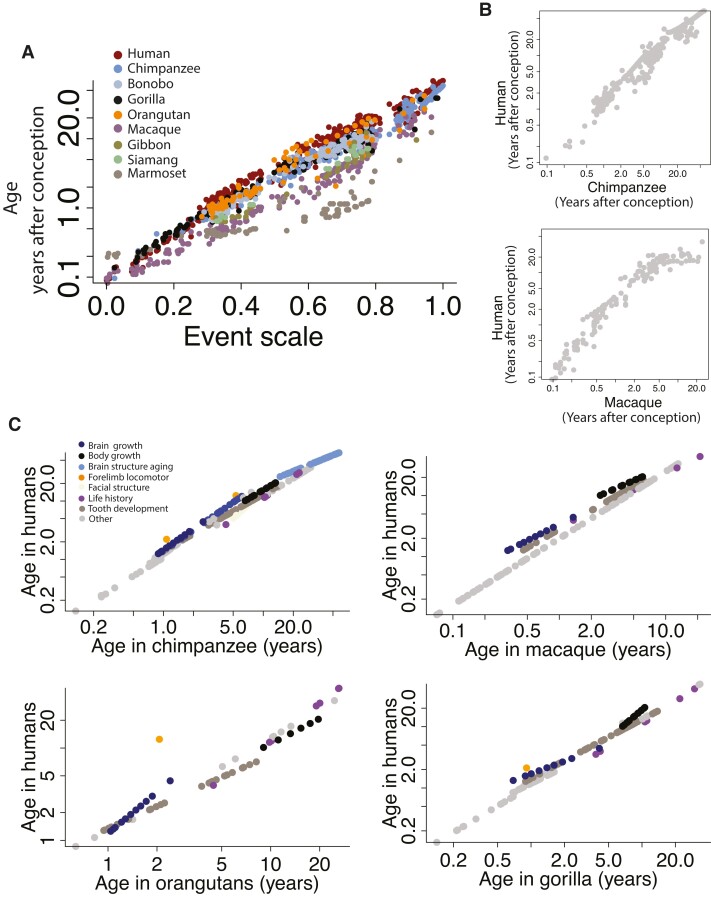
A) Predicted time points expressed in years after conception are log-transformed and plotted against an event scale (an ordering of time points) for different species in the study. These analyses show that corresponding ages between humans and great apes are similar early in development but that corresponding ages gradually diverge with age across these taxa. Macaques and marmosets proceed at a pace of development and aging that differs from great apes and humans. Time points in macaques occur earlier than they do in great apes and humans. B) We also include observed time points for two-species comparisons. C) Output of the model shows that some translated time points deviate relative to others.

**Fig. 5.**
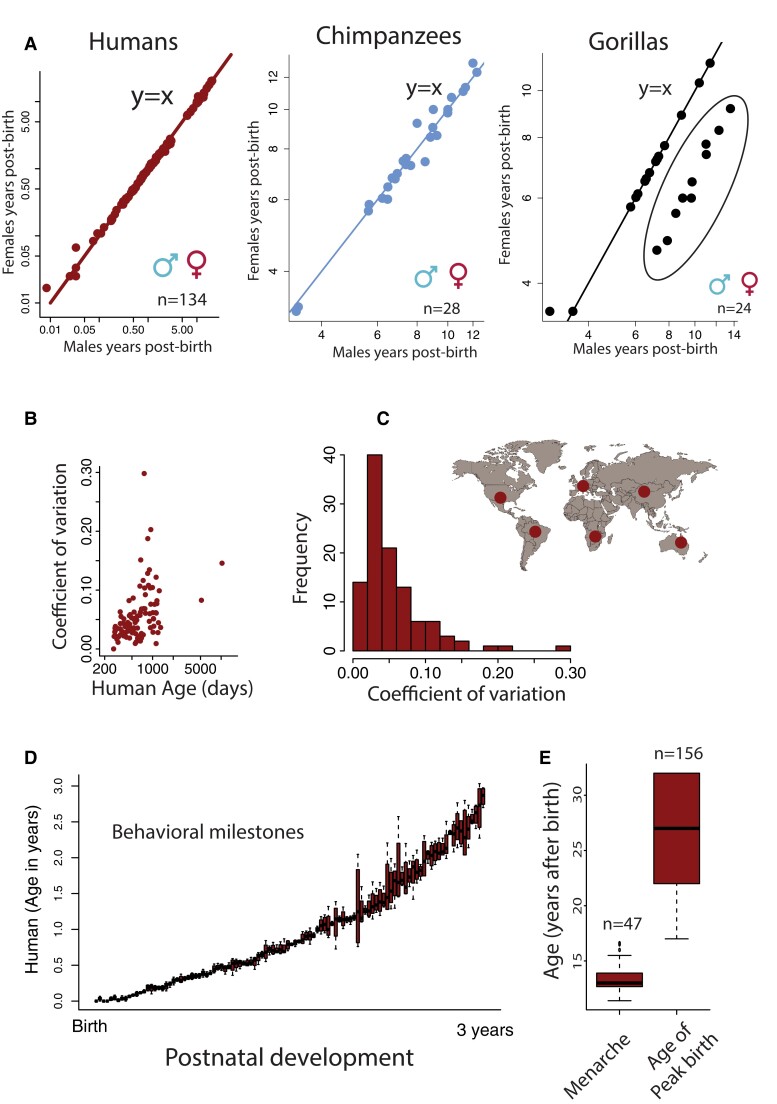
We considered individual variation (A–E) in collected time points in translated ages, including (A) sex differences. The pace of development is similar across males and females in humans, chimpanzees, and gorillas, with most time points close to *y* = *x*. A few time points deviate from others as is evident in gorillas where body growth is protracted in males versus females (circles encapsulated by a sphere). Overall, B) the coefficient of variation is similar across ages in humans though the standard deviation increases with age. This is evident when comparing locomotor milestones (D) across the first years of life and from the distributions in the age of peak birth versus menarche in humans.

**Fig. 6.**
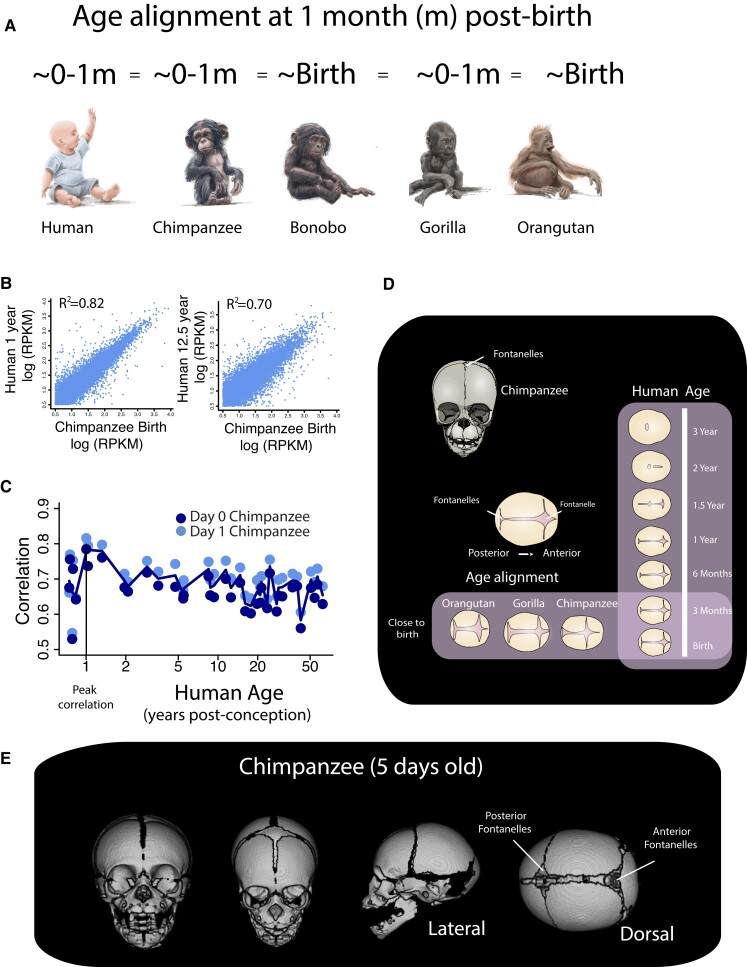
A) According to our model, humans and great apes are roughly similar in maturity around birth though there is within species variation. We also considered normalized gene expression B and C) and fontanelles (D) as an additional basis to align ages around birth. C) We correlated the log-transformed RPKM values of the frontal cortex of chimpanzees around birth (day 0 and 1 day after birth) with those of humans at different developmental ages. C and D) The correlation coefficients from tested associations between the log-transformed RPKM values between the frontal cortical areas in chimpanzees near birth (at day 0 and day 1) and those humans from different ages show that the frontal cortex of chimpanzees resembles humans that are slightly older than at birth. More precisely, newborn chimpanzees mostly align with humans at 1 year of age. D) We next evaluated great ape and human fontanelles. We drew fontanelles from the dorsal view of the skull CT scans. The posterior fontanelle recedes followed by the anterior fontanelles with age in humans. The anterior and posterior fontanelles of orangutan, gorilla, and chimpanzee individuals close to birth resemble the fontanelles of newborn humans, which suggest great apes most closely resemble humans around birth. E) CT scans are from the Digital Morphology Museum, Kyoto University Primate Research Institute. Anterior and posterior fontanelles are evident in this 5-day-old chimpanzee.

**Fig. 7.**
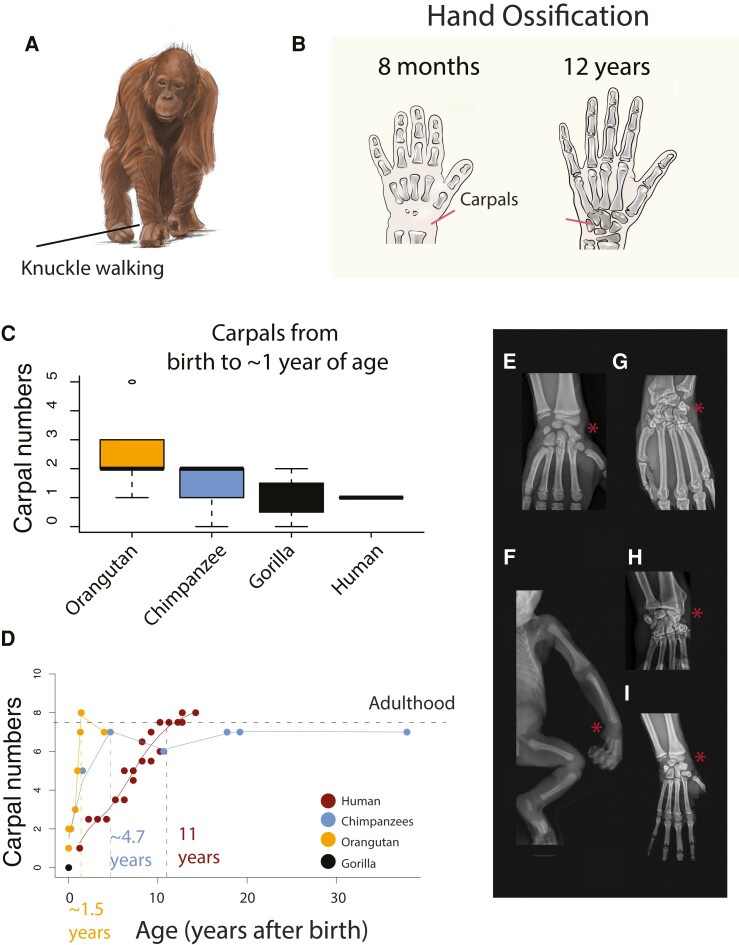
A) Great ape knuckle-walk (e.g., orangutans; A) but not humans. We focused on carpal maturation (B) because carpals are substrates for knuckle walking. C) We quantified ossified carpal numbers at birth across different species and over the course of postnatal maturation (D) from CT scans as well as radiographs from different great apes (E–I). Red asterisks highlight carpal bone ossification or lack thereof. C) Within the first year of life, some great apes have a higher number of ossified carpal bones than humans, but these differences are not statistically significant. Species differences become increasingly evident postnatally. D) Ossified carpal bone number increases at a much faster pace in great apes (i.e. orangutans and chimpanzees) than in humans. Adult carpal numbers are reached at approximately 1.5 years of age in orangutans, 4.7 years of age in chimpanzees, but 11 years of age in humans. We include some examples of radiographs from gorillas (E, I), including a newborn gorilla (F), and chimpanzees (G and H; 33). Drawings in (B) are from humans.

We considered 573 time points to align ages across 9 primate species (i.e. humans, chimpanzees, bonobos, orangutans, gorillas, rhesus macaques, gibbons, 2 siamangs species, and marmosets; Figs. 1–7; Figs. S1–S2; Tables S1–S9). Some of these are from transcriptional (Fig. 1D; S2–S7), anatomical (Fig. 1E, S8–S12), and behavioral variation (Fig. 1F), but they also specify sex (Fig. 5A) and include life history (Fig. 5B–D). Time points were obtained across pre- and postnatal periods. They include the age at which prenatal reflexes emerge and when neurons switch from a proliferative to a post-proliferative state. Postnatal time points were collected from abrupt and gradual changes in the brain, body, and behavior (Figs. 1–4). Most of the data are from individuals, but approximately 2% of these time points are from the organoids (n = 11; Figs. 3 and S7). We collected time points (e.g., age of menarche) from several human populations and from nonhuman primates living in different environments (i.e. wild or captive) to capture within-species variation (e.g., Figs. 5B–E and S1). Most of the time points are from captive individuals (Fig. S1B, D). Few equivalent time points were obtained across wild individuals so we refrained from testing the effects of the environment on the pace of development and aging. We considered sex differences within species (Fig. 5A, Table S1).

### Machine learning models generate age alignments from brain transcription

We used gene expression (Figs. 2–4 and S3–S7) and time points from anatomical variation (Figs. S8–S10) to extract corresponding time points that we used as time points in the translating time model. We tested 6 machine learning models (i.e. lasso and elastic-net regularized generalized linear models, support vector regression, k-nearest neighbor, random forest, Gaussian process regression) to predict ages across species. Anatomical variations include fractional anisotropy (Fig. S8), brain, and body growth (e.g., Figs. S11 and S12). We used three datasets, two of which consisted of normalized gene expression extracted from the frontal cortex of individuals varying in age (Figs. 1–4 and Table S1), and one of which consists of normalized gene expression from human and gorilla cerebral organoids also varying in age (n = 42; Figs. 4 and S7). Gene expression was normalized to transcripts per million. Additional details are in Table S2. These data consist of relatively few individuals with many sampled genes (10,000∼13,000). We selected 6 models that have made successful predictions with relatively small samples (20, 24–26,; Figs. 1,–3). Data were randomly partitioned into a training set (∼70% of the data) and a testing set (∼30% of the data; Figs. 2 and S13). Then, we used a measure of the difference between predicted and observed ages, called the root mean square error (RMSE), to assess age prediction accuracy (Figs. S5–S10). A similar approach was applied to the generation of cross-species age alignments from diffusion metrics (Fig. S8) and growth trajectories (Figs. S11 and S12).

For cross-species age alignments generated from transcription in tissues, the training set was drawn from humans as well as from humans and chimpanzees (Figs. 1–4). The integration of human and chimpanzee samples was used to increase the sample used to train the model. These datasets were selected to cover a wide age range. First, we translated age in chimpanzees to human age according to past work (19), and we trained these models to predict chronological age within species (Fig. S6). Overall, RMSE values were similar across models (Fig. 2E), but the Lasso and Elastic-Net Regularized Generalized Linear Models (glmnet R package), support vector regression, random forest, and Gaussian regression produced the lowest RMSE scores across datasets (Figs. S5–S10). We trained each model to predict age in humans. Subsequently, we imported normalized gene expression from species other than humans to predict age. Because the model had been trained to predict age in humans, the model translates the age of imported normalized gene expression from nonhuman primates to human age. Specifically, we used the ages of nonhuman primates and those translated to humans in the model.

Finally, we compared translated ages from machine learning models with past work (19, 27, 28). We log-transformed time points with age expressed in days after conception, and we fit a linear model to predict age in humans from the age of macaque time points and their square (R^2^ = 0.95; F = 1429; *P* < 0.01; Figs. 1 and 3 and S6; Fig. 1D), but the inclusion of RNA from our machine learning model as a factor does account for a significant percentage of the variance (estimate = 0.08 t = 2.987, *P* = 0.00325, R^2^ = 0.93). There is a strong overlap in extrapolated ages across methods, which supports the notion that they are valid to generate cross-species age alignments.

### Machine learning models generate age alignments from organoids

We used machine learning models to find corresponding ages across human and gorilla organoids (Figs. 3A, B, and S7) collected from 0 to 25 days post-incubation (Fig. 3C). We first trained the model to predict age in humans from gene expression. We then imported normalized gene expression in this trained model to predict age from gorilla organoids (Fig. 3D). Since the model had been trained to predict age in humans, inputting normalized gene expression from gorilla organoids predicts age in humans. We used the age of gorilla organoids and those translated to humans as corresponding time points in the model.

We compared cross-species age alignments from organoids with other metrics to determine how the use of organoids may apply to translating ages across species (Fig. 3B). We fit a linear model to log-transformed time points from organoids (n = 11) and individuals (n = 147) in humans and gorillas (with gorillas as the predictor variable). The model accounts for a significantly high percentage of the variance (*y* = 1.08*x* − 0.13; R^2^ = 0.97). The addition of tissue type (in vivo versus in vitro) accounts for a significant percentage of the variance (estimate = −0.11; t = −2.9; *P* = 0.004). Therefore, there are some differences in age alignments between organoids and individuals.

### A model to translate ages across the lifespan

We fit a general linear model to equate log-transformed corresponding ages across species. We first imputed the data because time points are not collected across all species (Fig. S14). Time points were averaged across species to generate averaged time points for each event. The event scale was computed by subtracting each time point by the earliest time point and dividing these values by the latest and earliest time point (Fig. S14). The event scale varies from 0 to 1 with early time points being assigned low scores and values close to 1 being assigned to high scores. We included predictors and factors in the model. These include the event scale, species, and event type. We use event type to test for heterochrony (i.e. modifications in the relative timing of biological processes) below. The inclusion of interaction terms permits testing for species differences in the relative speed of development and aging (i.e. variation in slopes in the model). We tested the following factors:


$$\begin{array}{rl} \mathrm{Age}= & \left(\mathrm{Event}\;\mathrm{scale})+(\mathrm{Species})+(\mathrm{Event}\;\mathrm{type}\right)\\ & +(\mathrm{Event}\;\mathrm{scale}*\mathrm{Species}*\mathrm{Event}\;\mathrm{type})+(\mathrm{Event}\;\mathrm{scale}{)}^{2} \end{array}.$$

**Fig. 8.**
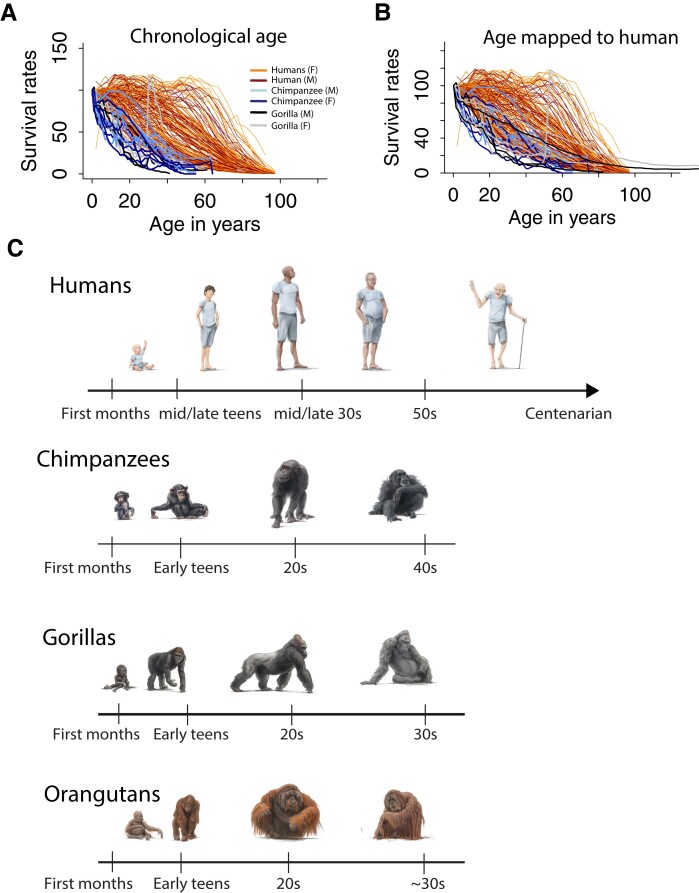
We captured survival rates across diverse human populations as well as humans and chimpanzees. Separate survival rates are generated for males and females. A) Survival rates are plotted against chronological age in humans and gorillas, which show that humans live overall longer than chimpanzees and gorillas in terms of absolute ages yet there are species differences in the pace of development and aging that should be controlled for. B) Age in chimpanzees and gorillas is translated onto humans to control for cross-species variation in the pace of development and aging. Accordingly, survival rates are shifted in humans relative to great apes after cross-species age alignments. C) Approximate age alignments across humans, gorillas, orangutans, and chimpanzees. Age translations at late stages of life are approximate, especially for gorillas and orangutans for which data to generate age alignments are sparse.

Age is expressed in log-transformed days after conception. This model accounts for a significantly large percentage of the variance (R^2^ = 98.8%; F = 876; *P* < 2.2e−16; Figs. 1 and 3) and captures the pace of development and aging across species. Early in development, corresponding ages are roughly similar across humans and great apes, but corresponding time points gradually diverge with age (Figs. 3 and 8). Humans and great apes are roughly similar in age in their first year of life, but a human in their mid-30 s equates to a chimpanzee in their mid-20 s and a gorilla in their early 20 s (Figs. 1 and 3, Fig. 8). The pace of aging is extended in humans compared with nonhuman primate species.

### Individual variation in cross-species age alignments

There is individual variation in the pace of development and aging. We collected time points across diverse human populations, sampled across Africa, Europe, Asia, and the Americas. We considered behavioral milestones, menarche, and age of peak births (populations: n = 4; 47; 156, respectively (29–31,; Fig.5). Variation in these biological and behavioral traits generally increase with age (Fig. 5C–E). The coefficient of variation (Fig. 5B), which is calculated for time points from the age of peak births, menarche, and behavioral milestones, extends up to 0.3 (5–95% CI: 0.01–0.13). We consider that this coefficient of variation captures plausible variation in extrapolated age alignments in humans and is to be used as a guide in extrapolating ages across species.

We tested for sex differences in species for which a total of 188 time points were available for comparison. The pace of development and aging is similar across the sexes (Fig. 5A) in humans (n = 134), chimpanzees (n = 28), and gorillas (n = 26). Many biological time points lie close to a *y* = *x* regression, which shows similarity in the pace of development across males and females. This is the case in humans (slope: 1.01; SE: 0.003; *y* = 1.01*log(*x*) − 0.02; R^2^ = 0.99), chimpanzees (slope: 0.94; SE: 0.033; *y* = 0.94*log(*x*) + 0.21, R^2^ = 0.97), and gorillas (slope: 0.99; SE: 0.1560; *y* = 0.99*log(*x*) + 0.07; R^2^ = 0.60. There is no obvious pattern where one sex takes longer to develop relative to the other across these three species. Notably, not all time points lie on a *y* = *x* regression (Fig. 5A). Some biological pathways occur for an unusually long time in one sex versus the other, as is the case for body growth trajectories, which are extended in male gorillas (Fig. 5A; Table S3, see sphere). Although some time points deviate from others across the sexes, males and females proceed through similar biological and behavioral trajectories.

### Testing accuracy of cross-species age alignments

We tested the accuracy of our model in equating corresponding ages across species. We used maturational states of humans and great apes close to birth because of the availability of samples near birth. According to the model, a human in their first year after birth roughly equates to great apes in their first year of life (Fig. 6A). We considered cortical transcriptional profiles (Fig. 6B) and cranial sutures (i.e. fontanelles; Fig. 6D–E) of humans and great apes close to birth.

We aligned ages based on transcriptional profiles from the frontal cortex of 38 humans and 2 chimpanzees (Fig. 6B and C) (32). We correlated the log-transformed reads per kilobase per million (RPKM) values of chimpanzees at days 0–1 post-birth with homologous genes in humans (n = 12,557) ranging in age from close to birth to approximately 62 years of age. Only genes with a minimum expression were considered in these analyses (log10 (RPKM) > 0.5). We found that the transcriptional profiles of chimpanzees around birth (days 0–1 post-birth) most strongly correlate with humans at 1 year of age (Fig. 6C), highlighting the similarity in the maturational state of humans and chimpanzees within the first year of life.

Next, we aligned ages based on the fontanelle maturity in humans and great apes. Human posterior and anterior fontanelles close at around 1–3 years (Fig. 6D). At birth, the anterior and posterior fontanelles extend across the medial to lateral axes of great apes and resemble that of human newborns (Fig. 6, S16, and S17). The pattern of anterior and posterior cranial fontanelles of gorillas, orangutans, and chimpanzees near birth resembles humans that are also within their first year of life (Fig. 6D). Collectively, these observations suggest that great apes and humans are similar in degree of maturity in their first year of life, which agrees with the results from our model.

### Extended duration of biological programs in humans

Although the model accounts for a significant percentage of the variance in translated ages, some biological processes (e.g., carpal bone ossification, brain growth, and locomotor behavior) deviate from others (Fig. 7) (33). Carpal ossification is one example where the timing of ossification is accelerated in great apes. We quantified ossified carpal numbers throughout development in humans and some great apes. At birth, carpal numbers are generally higher in great apes than in humans, but these species differences are not significantly different (ANOVA: F = 1.8; *P* = 0.197; n = 17; Fig. 7). Carpal bone ossification occurs at a much faster pace in great apes (i.e. orangutans and gorillas) than in humans. Ossified carpal bone numbers reach adult levels much earlier in orangutans (i.e. 1.5 years) and chimpanzees (i.e. 4.7 years) than they do in humans (11 years; Fig. 7). There are some sex differences in the rate of carpal ossification but both sexes reach adult numbers around 10–14 years of age (Fig. S18).

We classified each time point (i.e. brain growth, body growth, carpal ossification, organoid, cortical growth, and locomotor development) and tested for deviations in the timing of biological pathways (Tables S1–S6; Figs 3 and S1). The addition of body growth, brain growth, life history, structural aging, facial structure development, and locomotor forelimb development as factors in the model accounted for a significant percentage of the variance (Fig. 3). Therefore, there are significant heterochronies across species.

### Evolution of lifespan in human and nonhuman primates

We evaluated whether the human lifespan is extended relative to great apes after cross-species age alignments (Fig. 8). Our translating time model captures time points up to 68 years of age in humans, which corresponds to 57 years of age in chimpanzees, and their equivalent in other primate species. We calculated the relative number of individuals (e.g., 90%, 80%, and 70%) surviving up to a specific age (Fig. 8). Survival rates are extended in humans relative to great apes whether we align ages based on chronological ages (Fig. 8A) or map age in great apes onto human age (Fig. 8B). The extension in the human lifespan relative to great apes does overlap with some human populations but is clearly shifted relative to most human populations. These plots demonstrate that the human lifespan is extended relative to studied great apes.

## Discussion

We generated cross-species age alignments across humans and other primates. We identified which biological programs are conserved and which have been modified in humans. The inclusion of time points from diverse populations captures individual variation. One important finding from the present study is that the human lifespan is extended relative to great apes after cross-species age alignments.

### Age alignment across humans and nonhuman primates

This work expands on a long-term project called Translating Time (www.translatingtime.org), which relied on abrupt transformations to align ages during prenatal and early postnatal development in humans and model systems (18, 34). Here, we aligned biological pathways from abrupt and gradual changes in transcriptional, structural, and behavioral variation in order to find comparable ages across the lifespan of human and nonhuman primates. Some datasets rely on brain regions that may have human-specific features (e.g., prefrontal cortex [PFC]). Nonetheless, age alignments from the PFC generated age alignments that are comparable to other kinds of time points. We tested several machine learning techniques to find models best suited to generate cross-species age alignments. This integrative approach expanded our dataset by an order of magnitude relative to past studies (34, 35).

Our model aligns ages across species. Early in development, corresponding ages are similar across humans and great apes but they gradually diverge with age (Fig. 8). For instance, a human in their first year of age equates to a gorilla, orangutan, bonobo, and chimpanzee at roughly similar ages but cross-species differences in the pace of development and aging become salient with age (Fig. 8). Our study focused on translating ages at the individual level and not specific biological processes or organs. We plan to expand the database further to translate ages from specific organs.

Capturing information across diverse populations can be used to quantify variation in translated ages. Environmental factors also impact the pace of development and aging, with noticeable differences between captive and wild populations. We captured time points from diverse human populations, and from great apes, many of which were held in captivity. The results from our study mostly apply to captive primates. One notable observation is that the pace of development and aging are similar, but a subset of time points deviates from most others within each species (Fig. 5). Our dataset only captures age ranges up to mid-teens in great apes. We have yet to evaluate potential variation in the pace of aging in these species and investigate these sex differences. We plan to do so in future studies.

### Modifications in carpal ossification: an example of heterochrony

We found that some time points were protracted relative to other time points in some species. For example, the rate of carpal bone ossification is accelerated in great apes relative to humans and is likely linked to species differences in locomotion. Knuckle walking, which is specific to great apes, and our study shows that species differences in these forelimb locomotor adaptations are linked to carpal bone ossification acceleration. The presence of heterochronies like this among species, calls for the need to carefully select adequate time points for age alignment depending on the tissue/system under investigation.

### Old age as a distinctively human feature

Our findings demonstrate variation in the pace of development and aging across species. It takes longer for humans to proceed through biological processes than it does in nonhuman primates (Fig. 1A). Importantly, the human lifespan is unusually extended compared to great apes. This is true after accounting for variation in the pace of development and aging across species. It is rare for chimpanzees to live beyond 40 s years of age (36–39) and for marmosets to live beyond 10 years of age (40). These ages in chimpanzees and marmosets equate roughly to humans in their 50 s. Menopause occurs around age 40–50 s in humans. Accordingly, menopause should occur towards the end of the lifespan in marmosets as in chimpanzees. Menopause has been rarely observed in a few chimpanzees and elusive in marmosets (40, 41, 42). Although we cannot discount the possibility that menopause or other biological processes are human specific, our working hypothesis predicts that extending the lifespan in nonhuman primates should reveal biological processes that occur at late stages (e.g., menopause, brain atrophy, brain plaques, and tangles).

Lifespans are malleable, and enhanced care of great apes could lengthen lifespans. We suggest that the extension in human lifespan explains species differences in biological pathways in old age. The shorter lifespan of great apes relative to humans may explain the difficulties in observing plaques, tangles, and brain atrophy in great apes (39–43,–44). Extending great ape lifespan may reveal biological processes, including pathologies that are currently thought to be unique to humans. However, it is hard to understand if the extension of human lifespan is a cause or a consequence for the human-specific biological pathways and disease.

In old age, humans and great apes suffer from both similar and nonoverlapping diseases, which may contribute to species differences in lifespan (44,45). Chimpanzees and humans suffer from heart diseases, but chimpanzees are more likely to suffer from interstitial myocardial fibrosis, whereas humans are more likely to suffer from coronary–artery atherosclerosis (46, 47). Moreover, humans are more likely to suffer from cancer relative to chimpanzees (44, 48), but chimpanzees are more likely to die of viruses and bacterial infections than humans. Information on disease incidence in great apes is from captive records. Disease incidence is likely heavily impacted by environmental factors, including stress and diet, and the relative disease incidence likely varies between wild and captive populations of great apes.

### Translating time in organoids opens new avenues for comparative research

Brain organoids are becoming useful tools to model physiology in recent years. Organoids have increasingly been used to investigate molecular differences in the development across species where the acquisition of tissue is particularly challenging, as is the case for great ape studies. Our data show very little difference in age alignments when comparing time points from an in vitro development (i.e. in organoids) from individuals (Fig. 4B). Therefore, organoids may be valid tools to perform comparative developmental studies across species. We did find some differences between organoids and individuals when translating ages across species. More comparisons are needed to evaluate similarities and differences in age alignments across organoids and individuals.

### Translating time in great apes enhances conservation efforts

The present study provides translational tools to find equivalent ages across the lifespan of humans and great apes (e.g., orangutans and gorillas). This work is expected to enhance our ability to detect abnormalities at early stages and improve the timeliness of interventions in both humans and nonhuman primates. The ability to track development in critically endangered species such as gorillas and orangutans, for which data on biological timelines are sparse, can improve treatment and increase the population size of these threatened species. The results from the present study can now be used as a baseline against which to detect typical developmental timelines in studied great apes.

## Conclusions

Our work builds a resource to align ages across humans, great apes, and monkeys. We identified which biological programs are conserved and which have become modified over the course of development and aging. Capturing these basic parameters across human and nonhuman primates can be used to enhance tracking capabilities in humans as well as in great apes.

## Materials and methods

We gathered time points from abrupt and gradual changes in transcription, anatomy, and behavior across 9 species (Figs. 1 and S1) and from diverse human populations (see Supplementary Appendix; Figs. 1 and 4; Table S1). Data were imputed to generate an event scale (Fig. S2; see Supplementary Appendix). Statistics were performed with the programming language R.

### Behavioral and structural variation for age alignments

We used bone radiographs, some of which were provided by the North Carolina Zoo. These data were used retrospectively, collected for purposes other than this study, and were approved for use by the North Carolina Zoo IACUC committee. We used these and other images to track carpal ossification maturation (Fig. 7E–I). We quantified the number of discernable ossified structures in the wrist (Figs. 7B and S18).

We extracted time points from peaks and plateaus in growth trajectories (Figs. 1–3). We fit nonlinear regressions through the data to extract the age at which individuals reach specific percentages of adult volumes (Figs. S11 and S12). We also fit nonlinear regressions to capture the age at which peaks in specific biological processes occur across different species (e.g., age of peak births; Fig. S15). Some data were extrapolated from the Web Plot digitizer. In some cases, data points may have been obscured by others on plots, or data collected from regressions. Therefore, the time points collected may vary slightly from that reported in the original study.

### Transcriptional variation for age alignments

We trained 6 machine learning models to predict corresponding ages from normalized gene expression from individuals at different ages (20, 32, 49–51). We also used human and gorilla organoids to generate cross-species age alignments (Fig. 5; see Supplementary Appendix). These data are from publicly available datasets (32, 50, 51).

## Supplementary Material

pgad230_Supplementary_Data

## Data Availability

Data are in the supplementary tables. Scripts are available on Dryad: (DOI: 10.5061/dryad.0cfxpnw7r). We obtained radiograph images from the North Carolina Zoo (contact email: research@nczoo.org). Further access to these data beyond supplied in the present study may require approval by the North Carolina Zoo.
