# Supplementary material for: Transcription, structure, and organoids translate time across the lifespan of humans and great apes
Source: PNAS Nexus. 2023 Jul 20;2(8):pgad230. doi: 10.1093/pnasnexus/pgad230 (PMC10406161; doi:10.1093/pnasnexus/pgad230)
Supplement: pgad230_Supplementary_Data [file pgad230_supplementary_data.zip › PNASNEXUS-PNASNEXUS-2022-01101R-s02.docx]

**Transcription, structure, and organoids translate time across the lifespan of humans and great apes**

Christine J. Charvet^1^, Kwadwo Ofori^2^, Carmen Falcone^3^, Brier A. Rigby-Dames^4,5^

^1.^ Department of Anatomy, Physiology and Pharmacology; College of Veterinary Medicine; Auburn University; Auburn, AL, USA

^2.^ Department of Biology, Delaware State University, Dover, DE, USA

^3.^ Department of Neuroscience, International School for Advanced Studies (SISSA), Trieste, Italy

^4.^ Department of Computer Science, University of Bath, Bath, UK

^5.^ Department of Psychology, University of Bath, Bath, UK

**Supplementary material**

We discuss how we filtered the genes for the application of machine learning models, how we imputed the data to generate the event scale, and the different sources that make up the dataset.

***Variation across populations in the wild and in captivity***

We collected time points from individuals in the wild and in captivity. We used menarche, age of peak births, and survival rates across diverse populations. We included survival rates from multiple sources from captive and wild populations. Survival rates were computed by quantifying the number of individuals at a particular age relative to individuals present at or close to birth. We only included survival rates from populations with a relative clear decline in the percentage of individuals^1,2^.

***RNA metrics for machine learning analyses***

We filtered genes with low average expression. Poorly expressed genes are typically noisier in their expression than highly expressed genes. We aimed to use a roughly similar number of genes sampled across datasets (~10,000 to ~13,000 genes). We performed analyses across humans and chimpanzees versus other nonhuman primates. We performed additional analysis where we extrapolated ages across humans and chimpanzees from normalized gene expression from the prefrontal cortex and included them in the dataset (Table S1, S2). For some tested models, there were missing values in resampled performance measures. We kept outputs of all six models across tested datasets to readily compare their performance across datasets.

***Imputation for the model generation***

We fit a linear model to translate ages. We imputed time points because data were not systematically available across all 8 studied species. Orangutans and gibbons were amongst the species for which data are sparse whereas data for humans and chimpanzees’ data are relatively complete (Fig. S1). We imputed missing data with imputation by linear regression through a prediction method implemented with a Monte Carlo simulation. We used the midas touch method, which imputes univariate missing data through predictive mean matching. We selected the imputed dataset with the highest correlation coefficients across individuals. Specifically, we evaluated the lowest correlation coefficient from each dataset, and we selected the dataset with the highest minimum correlation across individuals. We ordered these time points and subtracted each time point (averaged across species) by the minimum age and divided these values by the difference between the maximum and minimum average age. The ordering of time points varied from 0 to 1 with early time points assigned a score close to 0 and later time points assigned a score close to 1.  We collected the average time points across species with the weighted average proportional to the amount of missing data so that species with relatively fewer time points contributed less to the events scale than species with more complete data.

***Data source***

Time points are collected from multiple sources. Some time points were collected from multiple individuals, but other age alignments were collected from a single individual. Data on great apes are sparse, especially for highly endangered gibbons and orangutans (Fig. S1). We chose to maximize sample size even though sex and environmental conditions (i.e., captive versus wild) were not systematically known for every time point. We tagged time points from males versus females when this information was known (Fig. 5A and B). We include information about the environment and sex for the different individuals when possible. However, we were only able to collect very few time points per species (Fig. S1). We did not classify individuals by sex for prenatal and early postnatal time points because information about sex is frequently lacking from these developmental studies. We used multiple metrics where possible as well as large samples to overcome potential inconsistencies (Table S1). Despite these caveats, our approach resulted in an unprecedentedly large dataset and enables the translation of ages for highly endangered great ape species.

**Figure legends**

**Fig. S1.** We evaluate sample size and relative amount of missing data across species. (A) A barplot shows the number of time points collected for each species. More data were collected for humans and chimpanzees than for other species (e.g., gibbons, orangutans). We combined information for different subspecies of orangutans and gorillas due to the paucity of data available for orangutans and gorillas. (B) We did not distinguish time points for captive versus wild apes because of the paucity of data as is exemplified for chimpanzees (C) The data is largely composed of structural and behavioral time variation. (D) Most of the data are from captive rather than wild primate populations, and (E) most of the data is either averaged by sex or of unknown sex.

**Fig. S2.** (A-D) We imputed time points to generate an event scale. Examples are shown for (A) humans versus chimpanzees, (B) chimpanzees versus gorillas, and (C) bonobos versus orangutans. (D) Correlation coefficients from log-transformed time points for different species are higher for gorillas, chimpanzees, macaques, and humans (pink) than they are for gibbons and orangutans (blue). That is, gibbons and orangutans have the fewest time points and correlate least with other species.

**Fig. S3.** We used RNA sequencing datasets from multiple sources and from multiple species to generate cross-species age alignments (^3^(3); A-B), (^4^(4); C-D), and (^5^(5); E-F). (A, C, E) We performed a principal component analysis of log10-transformed normalized gene expression. (B, D, F) We also include histograms to include the age ranges for each dataset. Age here is expressed in years after birth of individuals. We used these data to test which machine learning models are best suited to align ages across human and non-human primate species.

**Fig. S4.** We compared corresponding ages extracted from machine learning models with other metrics to assess the accuracy of translated ages. (A) Corresponding time points extracted from machine learning models, other statistical procedures from a past study^6^, as well as other metrics (mostly structural and behavioral metrics) yield similar results. We fit a linear regression through the log-transformed values expressed in days after conception, which accounts for a significant and high percentage of the variance (93%), and this analysis is modified from a recent study^7^. (B) We also extracted time points from machine learning models across humans and gorillas. We found that time points from machine learning models align with other metrics. The linear regression across these data accounts for a significant and high percentage of the variance (96%). (C) We also used machine learning models to translate ages in gibbons. The time point extracted from machine learning models in gibbons appears to deviate from other time points.

**Fig. S5.** We used different models to compare age alignments from normalized gene expression extracted from the frontal cortex of humans, chimpanzees, gibbons, and gorillas with the root mean square error(A). (B-E) We considered how these models predict age in humans for which age is already known.

**Fig. S6.** (A) We used machine learning models to find corresponding ages between species. Here, we used one of these models to predict age from normalized gene expression from human, macaque, and chimpanzee frontal cortex. (B) Model selection was based on RMSE values (B), which compares the difference between actual and predicted ages. A low RMSE indicates good model performance. (C-E) We compared translated ages based on our machine learning models to our previous work collected^7,8^ to ensure the concordance of age alignments based on different methods. We mapped the age of chimpanzee and macaques onto humans (on the y axis) and we mapped ages of chimpanzees and macaques onto humans based on past work (x.axis^7,8^). Time points that lie in close proximity to the y=x regression demonstrate strong concordance across methods. Translated ages extrapolated from the lasso and elastic-net regularized generalized linear models (glmnet R package) generated best prediction accuracies.

**Fig. S7.** We tested 6 machine learning models to generate cross-species age alignments from human and gorilla organoids. (A) The glmnet R package, gauss poly, and SVR were considered the best models to translate ages because they produced the lowest RMSE values. Different models produced a range of corresponding ages across the two species. (B) We compared how different models yielded different translated ages in humans and gorillas. We generated a regression through human and gorilla time points from organoids and used this regression to extend ages across prenatal stages in humans and gorillas. (B) We included a y=x regression as a baseline to detect whether translated ages are similar in absolute days across these two species. That is, regressions that lie close to y=x mean that corresponding ages are similar across the two species. We selected translated ages produced by the gauss poly model. (C-H) Translated ages according to different models. The gauss poly (C), glmnet (D), and SVR model (E) were considered the best models based on RMSE values.

**Fig. S8.** We used fractional anisotropy, radial and mean diffusivity to align ages from machine learning models. (A) Fractional anisotropy varies similarly with age in humans and in chimpanzees. (B) We applied different machine learning models to extract corresponding ages from these metrics. (C) The random forest, lasso and elastic-net regularized generalized linear model, and support vector regression (SVR) produced the lowest RMSE scores. We therefore used the random forest model to align ages across the two species. Corresponding time points occur later in humans than in chimpanzees.

**Fig. S9.** (A) Age alignments generated from the growth of frontal cortical areas and the corpus callosum in humans and in chimpanzees. (B) We used the prefrontal cortex white matter volumes from humans and chimpanzees of different ages as one of multiple metrics to align ages across these two species. We first trained the model to predict age within a species, and relied on RMSE values as a basis for model selection. (C) RMSE values showed that the glmnet R package produced the lowest RMSE values. We therefore selected the glmnet to generate cross-species alignments. (D) We also include cross-species age alignments produced from different machine learning models for comparison. Corresponding time points occur later in humans than they do in chimpanzees.

**Fig. S10.** We aligned ages (A) from temporal growth metrics extracted from ultrasounds of fetal gorillas and chimpanzees. (B) We tested different machine learning models, and we selected the glmnet model based on RMSE values. Metrics used in the model include (C) include biparietal diameter (BPD), transverse abnormal diameter (TAD), humerus (HL), and femur length (FL). We fit smooth splines through the data in order to capture the same sample size across gorillas and chimpanzees. These analyses enable finding corresponding ages during fetal stages of development across great apes.

**
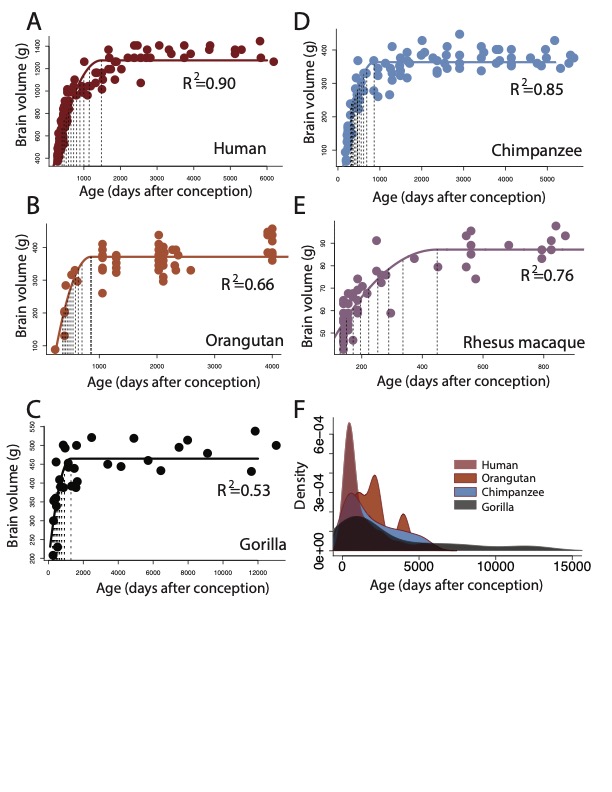
**

**Fig. S11.** We extracted time points from brain growth trajectories in humans (A), orangutans (B), gorillas (C), chimpanzees (D), and rhesus macaques (E^9^). We fit non-linear regressions (easynls, model=4) with age as the independent variable and brain volume (grams) as the dependent variable. We extrapolated the age at which the brain reaches adult volumes and percentages of adult volumes (vertical bars) from these non-linear regressions. These regressions capture when most of the brain ceases to grow but some, albeit small, growth may extend beyond these identified ages. Species differences in sample size may introduce variation in identified age of growth cessation across species. We, therefore, excluded time points that occur before 270 days after conception in humans and their corresponding ages in macaques (127 days after conception^10^) to capture similar age ranges across species. This is because brain growth appears accelerated at prenatal stages relative to postnatal ages in humans and macaques. Fetal data was not available for all studied primate species but their inclusion in a subset of studied species could impact the output of the regression. (F) Kernel density plots show the age ranges for available data.

**Fig. S12**. We include corresponding time points from body growth trajectories from great apes (A-D), lesser apes (E-F), and from monkeys (marmosets; G). Humans are also in the model. We fit a nonlinear regression (easynls, model=3) with weight versus age expressed in years. We fit regressions separately for males (triangles) and for females (circles). Sex differences are noticeable in some of the studied primates (e.g., gorillas, C; orangutans, D). We extracted ages at which the percentage of adult volume reaches a particular value (e.g., 100%, 90%, 80%) as a basis with which to equate corresponding ages across species. The dashed vertical lines represent time points extracted from these growth trajectories collected separately for males and females and are exemplified in chimpanzees (A). Not all groups reach adult weights. This is notably the case for male orangutans that grow throughout their life. We did not extrapolate time points for male orangutans. A greater age range is available for humans than for other studied non-human primates, which might skew the extrapolated time points. We therefore fit nonlinear regressions through weights for individuals from 2 to 34.5 years of age so that non-linear regressions are collected through similar age ranges across non-human primates. These data are from captive individuals.

F**ig. S13.** Overview of the approach used to generate cross-species age alignments from machine learning models. We first partitioned the data into a training and a testing set. The training set consisted of roughly 70% of the data, and the testing set consisted of the remaining 30% of the data. We trained 6 different models (e.g., random forest) to predict ages, and we checked the accuracy of these predictions. We use the root mean square error (RMSE) to select the best suited model. RMSE values measure the difference between actual and predicted values. We then generated cross-species age alignments by inputting data from one species model into these trained models.

**Fig S14.** Overview of pipeline used to generate an event scale. (A) Not every time point can be collected in every species. Some data are missing. Schematics show examples of missing versus measured time points for some species. We first imputed log-transformed time points (expressed in days after conception) to a dataset with no missing data. We then performed a weighted average of time points across species. Species with fewer time points carried less weight in the calculations than species with relatively large amounts of time points. (B) The event scale was generated by subtracting each time point by the earliest time point and dividing these values by the latest and earliest time point. The event scale varies from 0 to 1 with early time points being assigned low scores and values close to 1 being assigned to high scores. The event scale has been used in past studies to generate equivalent time points across species 7,8,10.

**Fig. S15.** We considered individual variation in the age of peak births across human populations across 156 countries as well as from hunter-gatherers. We also include the age of peak births for some non-human primates (i.e., chimpanzees, gorillas, orangutans, macaques, marmosets). (A) We considered the age of peak births from hunter-gatherers (B; n=5), and from industrialized societies. (C) We include a few examples, which include birth rates from American Samoa, Australia, and Botswana, to exemplify variation across diverse human populations. The 95% confidence intervals in the age of peak births range between 22 to 32 years of age in humans with a similar median average age between hunter-gatherers and industrialized societies. (D) We obtained the age of peak births from great apes (e.g., orangutans, gorillas, chimpanzees) and monkeys (e.g., rhesus macaques, marmosets^11^). The 95% confidence intervals in the age of peak births in non-human primates range between 4 (marmosets) to 25 years of age (gorillas). The y axis shows the mean number of births per female in a given interval of time and are shown as a percentage. (E) Age of peak births can vary dramatically based on environment as evident between captive and wild orangutans.


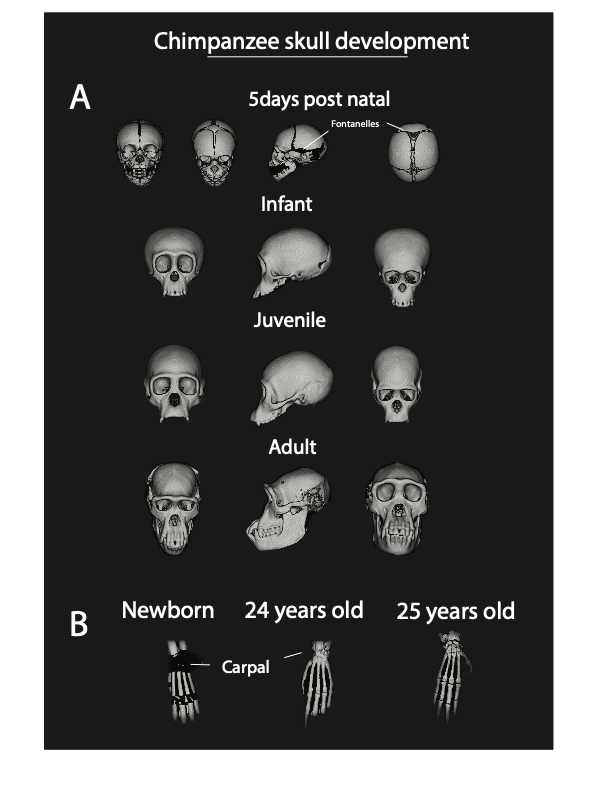


**Fig. S16.** We used CT scans of a chimpanzee, gorilla, and an orangutan estimated to have died close to birth. We use the maturity of key phenotypic traits (e.g., fontanelles, carpal bone ossification) as a complimentary approach to align ages across humans and apes to align ages across birth. We considered the presence of fontanelles and ossified carpal bones in great apes and in humans to align ages and detect possible deviations in the timing of biological pathways. According to the scans of these individuals, fontanelles have yet to close in these specimens, and this is true of the gorilla, orangutan, and the chimpanzee. In addition, carpals have yet to be ossified. These scans are from the visible ape project^12^ .

**Fig. S17.** We considered skull (A) and wrist maturation (B) from CT scans of chimpanzees from the Digital Morphology Museum, Kyoto University Primate Research Institute (KUPRI). Fontanelles are evident in the 5-day old newborn chimpanzee but not in the infant. Infancy is typically defined as ranging between birth to about 3 years of age^8^. According to these observations, fontanelle closure should occur somewhere between 0 to 3 years of age. Similarly, carpal bone ossification (B) extends postnatally. This is evident given that the 5-day old chimpanzee has many carpals that have yet to ossify.

**Fig. S18.** We compared male and female carpal ossification rates in humans. Despite the variation across males and females, carpal ossification is largely complete by 10 to 14 years of age in both sexes. Drawings represent carpal ossification from radiographs of humans at different postnatal ages in male and females in humans and are modified from radiographic atlas^13^. There are roughly 2 ossified carpals near birth, and this number steadily but slowly grows postnatally up to around ~12 years of age in humans in males as in females. Abbreviations: MO: months; YO: years. These qualitative observations align with quantitative comparisons (Fig. 7).


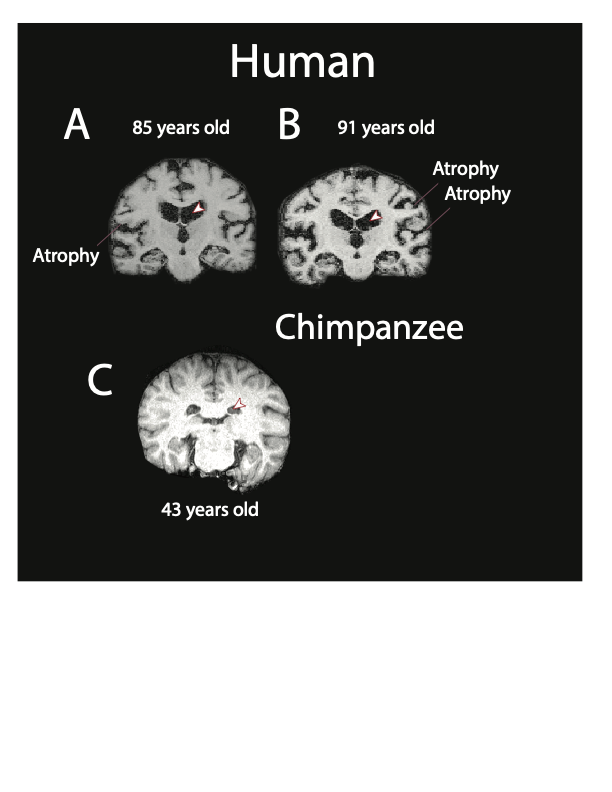


**Fig. S19.** Coronal slices through structural MR scans of aged human and chimpanzee brains show that human brains of 85 and 91 years of age are atrophied, especially at 91 years of age. This is in contrast with aged chimpanzees that do not possess obvious atrophy as exemplified by a 43-year-old chimpanzee. Ventricles (arrowheads) are expanded in aged humans In contrast, these structural modifications with age are not as evident in the aged chimpanzees. Chimpanzee and human structural MR scans are from the National Chimpanzee Brain Resource and the open access series of imaging studies (OASIS) database, respectively^14^.

**Supplementary tables**

**Table S1.** Time points used to find corresponding ages across primate species.

**Table S2.** Details of RNA sequencing datasets (e.g., sample size, age ranges).

**Table S3.** Data used for body growth trajectories across primate species.

**Table S4.** Data used for brain growth trajectories across primate species.

**Table S5.** List of specimen sources used to capture carpal bone ossification in primate species.

**Table S6.** Carpal numbers in different primate species.

**Table S7.** Age of menarche across human populations and great apes.

**Table S8.** Time points classified by sex.

**Table S9**. Summary statistics from the translating time model.

**SI References**

1. United Nations Department of Economic and Social Affairs. *United Nations Demographic Yearbook* (United Nations Department of Economic and Social Affairs, New York, 2019).

2. Colchero, F. *et al.* The long lives of primates and the 'invariant rate of ageing' hypothesis. *Nat. Commun.* **12,** 3666 (2021).

3. Liu, X. *et al.* Disruption of an evolutionarily novel synaptic expression pattern in autism. *PLoS Biol.* **14,** e1002558 (2016).

4. Benito-Kwiecinski, S. *et al.* An early cell shape transition drives evolutionary expansion of the human forebrain. *Cell* **184,** 2084-2102.e19 (2021).

5. Xu, C. *et al.* Human-specific features of spatial gene expression and regulation in eight brain regions. *Genome Res.* **28,** 1097-1110 (2018).

6. Zhu, Y. *et al.* Spatiotemporal transcriptomic divergence across human and macaque brain development. *Science* **362,** eaat8077 (2018).

7. Charvet, C. J. *et al.* Tracing modification to cortical circuits in human and nonhuman primates from high-resolution tractography, transcription, and temporal dimensions. *J. Neurosci.* **42,** 3749-3767 (2022).

8. Charvet, C. J. Cutting across structural and transcriptomic scales translates time across the lifespan in humans and chimpanzees. *Proc. Biol. Sci.* **288,** 20202987 (2021).

9. Creel, J. *Ontogenetic Changes in Orangutan Brain Evolution: Is it Heterochrony? Doctoral Dissertation* (Texas Tech University, Lubbock, TX, 2012).

10. Workman, A. D., Charvet, C. J., Clancy, B., Darlington, R. B. & Finlay, B. L. Modeling transformations of neurodevelopmental sequences across mammalian species. *J. Neurosci.* **33,** 7368-7383 (2013).

11. Caro, T. M. *et al.* Termination of reproduction in nonhuman and human female primates. *Int. J. Primatol.* **16,** 205-220 (1995).

12. Barger, N., Martín, J. S., Boyle, E. K., Richmond, M. & Diogo, R. The visible ape project: a free, comprehensive, web-based anatomical atlas for scientists and veterinarians designed to raise public awareness about apes. *Evol. Anthropol.* **30,** 160-170 (2021).

13. Gilsanz, V. & Ratib, O. *Hand Bone Age: A Digital Atlas of Skeletal Maturity* (Springer, Berlin, 2012).

14. Marcus, D. S., Fotenos, A. F., Csernansky, J. G., Morris, J. C. & Buckner, R. L. Open access series of imaging studies: longitudinal MRI data in nondemented and demented older adults. *J. Cogn. Neurosci.* **22,** 2677-2684 (2010).
